# Unveiling the role of tRNA-derived small RNAs in MAPK signaling pathway: implications for cancer and beyond

**DOI:** 10.3389/fgene.2024.1346852

**Published:** 2024-03-26

**Authors:** Qurui Wang, Qinyuan Huang, Xiaowei Ying, Jinze Shen, Shiwei Duan

**Affiliations:** ^1^ Key Laboratory of Novel Targets and Drug Study for Neural Repair of Zhejiang Province, School of Medicine, Hangzhou City University, Hangzhou, Zhejiang, China; ^2^ Department of Clinical Medicine, School of Medicine, Hangzhou City University, Hangzhou, Zhejiang, China

**Keywords:** tRNA-derived small RNAs (tsRNAs), MAPK signaling pathway, cancer, non-cancer diseases, biomolecular regulation

## Abstract

tRNA-derived small RNAs (tsRNAs) are novel small non-coding RNAs originating from mature or precursor tRNAs (pre-tRNA), typically spanning 14 to 30 nt. The Mitogen-activated protein kinases (MAPK) pathway orchestrates cellular responses, influencing proliferation, differentiation, apoptosis, and transformation. tsRNAs influence the expression of the MAPK signaling pathway by targeting specific proteins within the pathway. Presently, four MAPK-linked tsRNAs have implications in gastric cancer (GC) and high-grade serous ovarian cancer (HGSOC). Notably, tRF-Glu-TTC-027 and tRF-Val-CAC-016 modulate MAPK-related protein expression, encompassing p38, Myc, ERK, CyclinD1, CyclinB, and c-Myc, hindering GC progression via MAPK pathway inhibition. Moreover, tRF-24-V29K9UV3IU and tRF-03357 remain unexplored in specific mechanisms. KEGG analysis posits varied tsRNAs in MAPK pathway modulation for diverse non-cancer maladies. Notably, high tRF-36-F900BY4D84KRIME and tRF-23-87R8WP9IY expression relates to varicose vein (VV) risk. Elevated tiRNA-Gly-GCC-001, tRF-Gly-GCC-012, tRF-Gly-GCC-013, and tRF-Gly-GCC-016 target spinal cord injury (SCI)-related brain-derived neurotrophic factor (BDNF), influencing MAPK expression. tRF-Gly-CCC-039 associates with diabetes foot sustained healing, while tRF-5014a inhibits autophagy-linked ATG5 in diabetic cardiomyopathy (DCM). Additionally, tsRNA-14783 influences keloid formation by regulating M2 macrophage polarization. Upregulation of tRF-Arg-ACG-007 and downregulation of tRF-Ser-GCT-008 are associated with diabetes. tsRNA-04002 alleviates Intervertebral disk degeneration (IDD) by targeting PRKCA. tsRNA-21109 alleviates Systemic lupus erythematosus (SLE) by inhibiting macrophage M1 polarization. The upregulated tiNA-Gly-GCC-002 and the downregulated tRF-Ala-AGC-010, tRF-Gln-CTG-005 and tRF-Leu-AAG-001 may be involved in the pathogenesis of Lupus nephritis (LN) by affecting the expression of MAPK pathway. Downregulation of tsRNA-1018, tsRNA-3045b, tsRNA-5021a and tsRNA-1020 affected the expression of MAPK pathway, thereby improving Acute lung injury (ALI). This review comprehensively dissects tsRNA roles in MAPK signaling across cancers and other diseases, illuminating a novel avenue for translational medical exploration.

## 1 Introduction

MAPK, a pivotal protein responsive to cellular cues, shuttles from the cell surface to the nucleus (Lee et al., 2020). This protein engenders the MAPK signaling pathway, a conduit receptive to G-proteins. This pathway orchestrates a three-tiered kinase cascade—MAPK, MAPKK, and MAPKKK—interlinked with pivotal cellular processes: proliferation, differentiation, survival, apoptosis, and transformation (Lee et al., 2020; Qin et al., 2021; Qin et al., 2012; Reschke and Afrin, 2022). Notably, the triumvirate of paramount routes encompasses the ERK pathway, c-Jun N-terminal kinase (JNK) pathway, and p38 pathway (Lee et al., 2020; Schett et al., 2000).

In our study, we introduced three levels of the MAPK signaling pathway: MAPK, MAPKKs, and MAPKKKs. Within the MAPK pathway, key G proteins such as RAS, RAC, CDC42, and RHO receive external signals and relay them to the MAPKKKs. These MAPKKKs, namely, RAF, MEKK, and TAK, correspond to the ERK pathway, JNK pathway, and p38 pathway, respectively. At the next level, MAPKKKs act as phosphokinase, stimulating the phosphorylation of MAPKKs. The MAPKKs include the MEK family and MKK family, comprising MEK1 and MEK2 for the ERK pathway, MKK4 and MKK7 for the JNK pathway, and MKK3 and MKK6 for the p38 pathway. Upon activation, MAPKKs target the MAPKs hierarchy, including ERK, JNK, and p38, leading to their phosphorylation and subsequent involvement in signaling pathways. This phosphorylation process influences downstream factors in the nucleus, thus modulating cellular responses (Fang and Richardson, 2005; Guo et al., 2020; Garcia-Hernandez et al., 2021).

The expression levels of tsRNAs are commonly evaluated through high-throughput sequencing technology. This comprehensive process includes RNA extraction, library construction, RNA sequencing, and subsequent bioinformatics analysis to derive the expression profile of tsRNAs (Saikia et al., 2014).

Concerning the subcellular localization of tsRNAs, while they are primarily known to function within the cytoplasm, their exact subcellular localization can vary based on their functions and origins. This variation necessitates specific experimental approaches for precise detection and analysis (Jiang et al., 2021).

**FIGURE 1:**
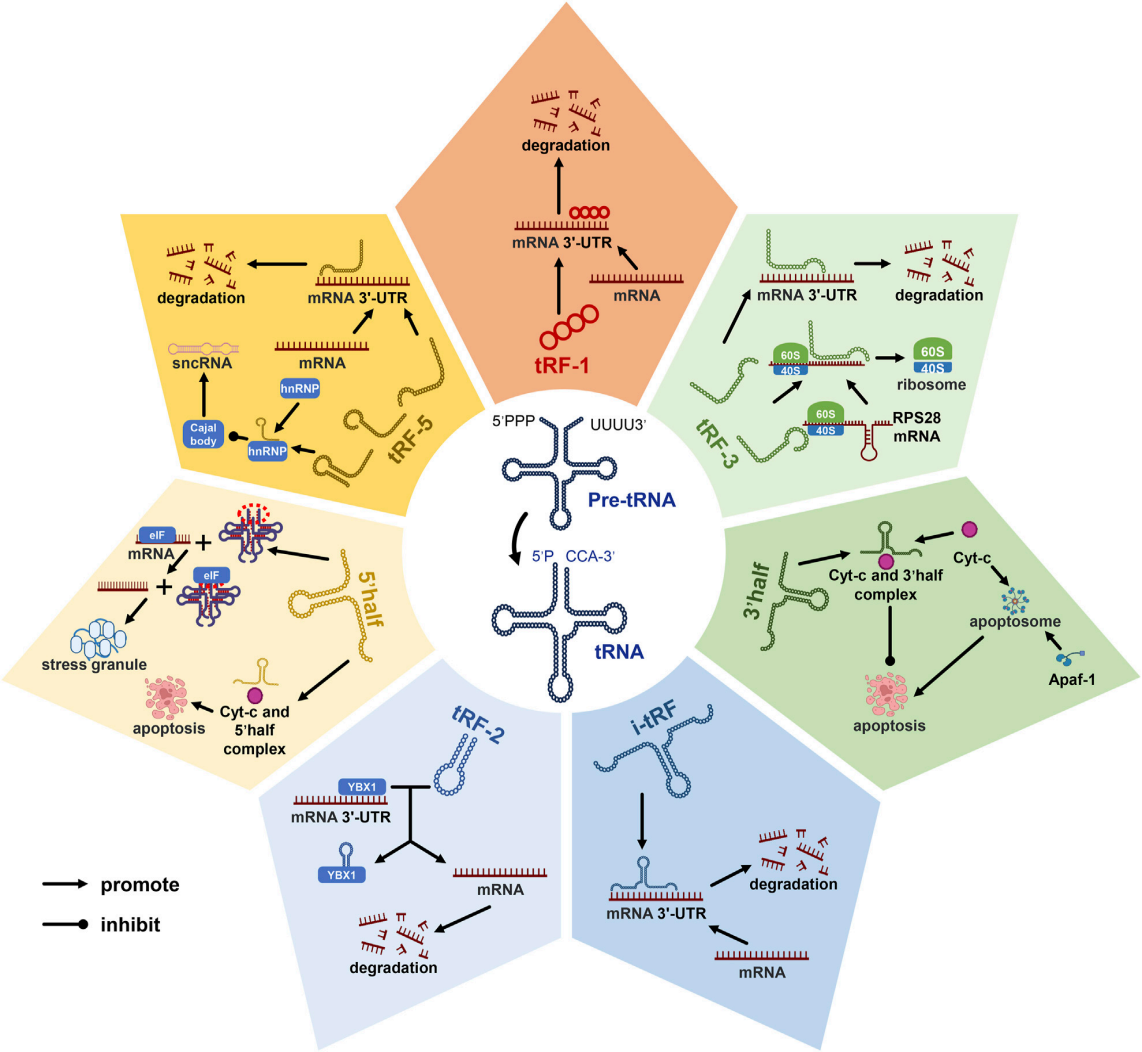
Diverse roles of tRNA-derived small RNAs (tsRNAs) in gene regulation. tsRNAs can be categorized into seven distinct subclasses based on the location of their source fragments within tRNA molecules, known as tRF-1, tRF-2, tRF-3, tRF-5, 5’half, 3’half, and i-tRF. Among these subclasses, tRF-1, tRF-3, tRF-5, and i-tRF have been identified as pivotal players in the transcriptional process, where they exert a suppressive influence on mRNA transcription. Additionally, tRF-3, tRF-5, 5’half, and 3’half tsRNAs have demonstrated their capacity to modulate gene expression at the post-transcriptional level. These findings shed light on the multifaceted roles of tsRNAs in cellular processes. Notably, in the context of this discussion, abbreviations such as Cyt-c (cytochrome c), mRNA (messenger ribonucleic acid), and sncRNA (small non-coding RNA) represent key molecular components, while pre-tRNA signifies precursor tRNA, and tsRNAs denote tRNA-derived small RNAs.tRF-2 engages Y-box binding protein 1(YBX1), displacing the 3′-UTR and stymying proliferation and metastasis.

Novel among small non-coding RNAs, tsRNAs, derived from mature or pre-tRNAs, span 14 to 30 nucleotides (Mao et al., 2023). These tsRNAs diverge into tRNA-derived fragments (tRFs) and tRNA-derived stress-induced RNAs (tiRNAs) (Gao et al., 2022; Chen and Shen, 2021; Chu et al., 2022). The manifold subclasses of tsRNAs, categorized based on their tRNA source fragment localization (Figure 1), encompass tRF-1, tRF-2, tRF-3, tRF-5, and i-tRF within tRFs, as well as tiRNA 5’half and 3’half (Chu et al., 2022). Functional duality characterizes tsRNAs, regulating both transcription and post-transcription events (Yu et al., 2020). For instance, tRF-1, tRF-3, tRF-5, and i-tRF mimic miRNAs or siRNAs, downregulating gene translation by binding target mRNAs (Pekarsky et al., 2016; Meseguer et al., 2019; Yu et al., 2020; Yu et al., 2021). Leveraging Cajal bodies, tRF-5 modulates diverse sncRNA production by binding heterogeneous nuclear ribonucleoprotein (hnRNP) (Boskovic et al., 2020). The formation of an RNA G-quadruplex by 5′-half replaces eukaryotic initiation factors, promoting stress granule assembly and concomitantly suppressing translation (Yamasaki et al., 2009; Ivanov et al., 2011; Lyons et al., 2016; Wang et al., 2020). Notably, tRF-3 interacts with the mRNA secondary domain of ribosome synthesis-related protein RPS28, promoting translation and augmenting ribosomal count (Yu et al., 2021). In concert, 5’half and 3’half bind cytochrome c (Cyt c), curbing apoptosome formation and activity (Saikia et al., 2014). Moreover, tRF-2 engages Y-box binding protein 1(YBX1), displacing the 3′-UTR and stymying proliferation and metastasis (Chu et al., 2022).

As illustrated below, considering the diverse regulatory mechanisms employed by tsRNAs in protein expression, we hypothesize that tsRNAs might exert their influence either by directly interfering with the molecular constituents of the MAPK pathway or by interacting with associated proteins. Through these mechanisms, tsRNAs could potentially impact the biological functions of cells. tsRNAs pivotal role unfolds in both the MAPK pathway and oncogenesis, as burgeoning research delves into their involvement in human ailments. Intriguingly, a comprehensive exploration of the interplay between the tsRNAs/MAPK axis and the genesis and progression of human diseases remains conspicuously absent.

This article fills this gap by systematically encapsulating current insights into the tsRNAs/MAPK nexus in disorders. Furthermore, it discerns present research limitations, charting a course for in-depth exploration and offering a cornerstone for future investigations.

## 2 MAPK-related tsRNAs in gastric cancer and spinal cord injury

In normal cells, the MAPK pathway transmits external signals through a series of kinase cascade reactions to regulate cell physiological functions. However, cells in many cancers will experience abnormal activation of the MAPK pathway, causing cells to be unable to respond correctly to external signals, thereby promoting the growth, proliferation and invasion of malignant cells (Jiang et al., 2021). Abnormal activation of the MAPK pathway has been observed in a variety of cancers, including breast cancer, colorectal cancer, lung cancer, etc [32046099].

In recent years, research has highlighted the significant regulatory role of tsRNAs in cancer (Zong et al., 2021). On the one hand, tsRNAs actively participate in regulating crucial physiological processes within cancer cells, including proliferation, differentiation, and apoptosis (Zhang et al., 2020). For instance, certain tsRNAs can function as competitive endogenous RNAs (ceRNAs), effectively competing with miRNAs to regulate the expression of genes associated with tumorigenesis (Zhang et al., 2020). On the other hand, abnormal expression patterns of tsRNAs are closely linked to the initiation and progression of tumors (Zong et al., 2021). Several studies have indicated that alterations in the expression levels of specific tsRNAs in cancerous tissues may correlate with the invasiveness, metastatic potential, and drug resistance of cancer cells (Zong et al., 2021).

As delineated in Table 1, a triad of MAPK-associated tsRNAs have been substantiated as pertinent to both Gastric Cancer (GC) and Spinal Cord Injury (SCI) (Qin et al., 2019; Dong et al., 2020; Xu et al., 2021; Xu W. et al., 2022). Rescuing assays conducted on the GC cell line HGC-27 unveiled the capacity of tRF-Glu-TTC-027 to modulate p-p38 and c-Myc (Xu et al., 2021). Complementary findings from experiments involving another GC cell line, NCI-N87, illustrated its potential to impact MAPK pathway-associated proteins, including p-ERK, p-p38, and c-Myc (Xu et al., 2021). In a parallel exploration utilizing fluorescent *in situ* hybridization (FISH) on GC cell lines HGC-27 and NCI-N87, tRF-Val-CAC-016 was identified as targeting MAPK pathway-related proteins, namely, CyclinD1, CyclinB, and c-Myc (Xu W. et al., 2022). Evidently, both tRF-Glu-TTC-027 and tRF-Val-CAC-016 exert their inhibitory effects on GC advancement through selective targeting of key proteins within the MAPK signaling cascade, thereby stifling MAPK pathway activity (Xu et al., 2021; Xu W. et al., 2022).

**TABLE 1:** Role of MAPK-related tsRNAs in human diseases and its characteristics.

tsRNAs	Human diseases	Effect of high tsRNA in human diseases	Clinicopathologicalcharacteristics	Ref.
tRF-Glu-TTC-027	Downregulated in GC	proliferation↓, migration↓, and invasion↓	low expression of tRF-Glu-TTC-027 is associated with a bigger tumor size and poor-differentiated histology	Xu et al. (2021)
		in GC cell lines (NCI-N87 and HGC-27)		
		tumor growth↓		
		in NCI-N87 xenografts mice		
tRF-Val-CAC-016	Downregulated in GC	proliferation↓	low expression of tRF-Val-CAC-016 is associated with a bigger tumor size and poor-differentiated histology	Xu et al. (2022a)
		in GC cell lines (NCI-N87 and HGC-27)		
		tumor growth↓		
		in NCI-N87 xenografts mice		
tiRNA-Gly-GCC-001, tRF-Gly-GCC-012, tRF-Gly-GCC-013, and tRF-Gly-GCC-016	Upregulated in SCI	—	—	Zhang et al. (2019)

GC, gastric cancer; SCI, spinal cord injury; tsRNAs, tRNA-derived small RNAs; tRFs, tRNA-derived fragments.

In the context of SCI, a meticulous analysis contrasting six rats with spinal cord contusion against control group of six rats spotlighted the heightened expression of tiRNA-Gly-GCC-001 in rat SCI (Qin et al., 2019). In-depth scrutiny involving Luciferase Assay and corroborative experiments validated the consequential impact of tiRNA-Gly-GCC-001 (Qin et al., 2019). This impact, wrought by the direct targeting of the 3′UTR of BDNF, was found to indirectly modulate MAPK levels, manifesting a discernible RNA silencing effect (Qin et al., 2019).

## 3 Exploring MAPK-related tsRNAs in disease: identification, expression, and pathway associations

**FIGURE 2:**
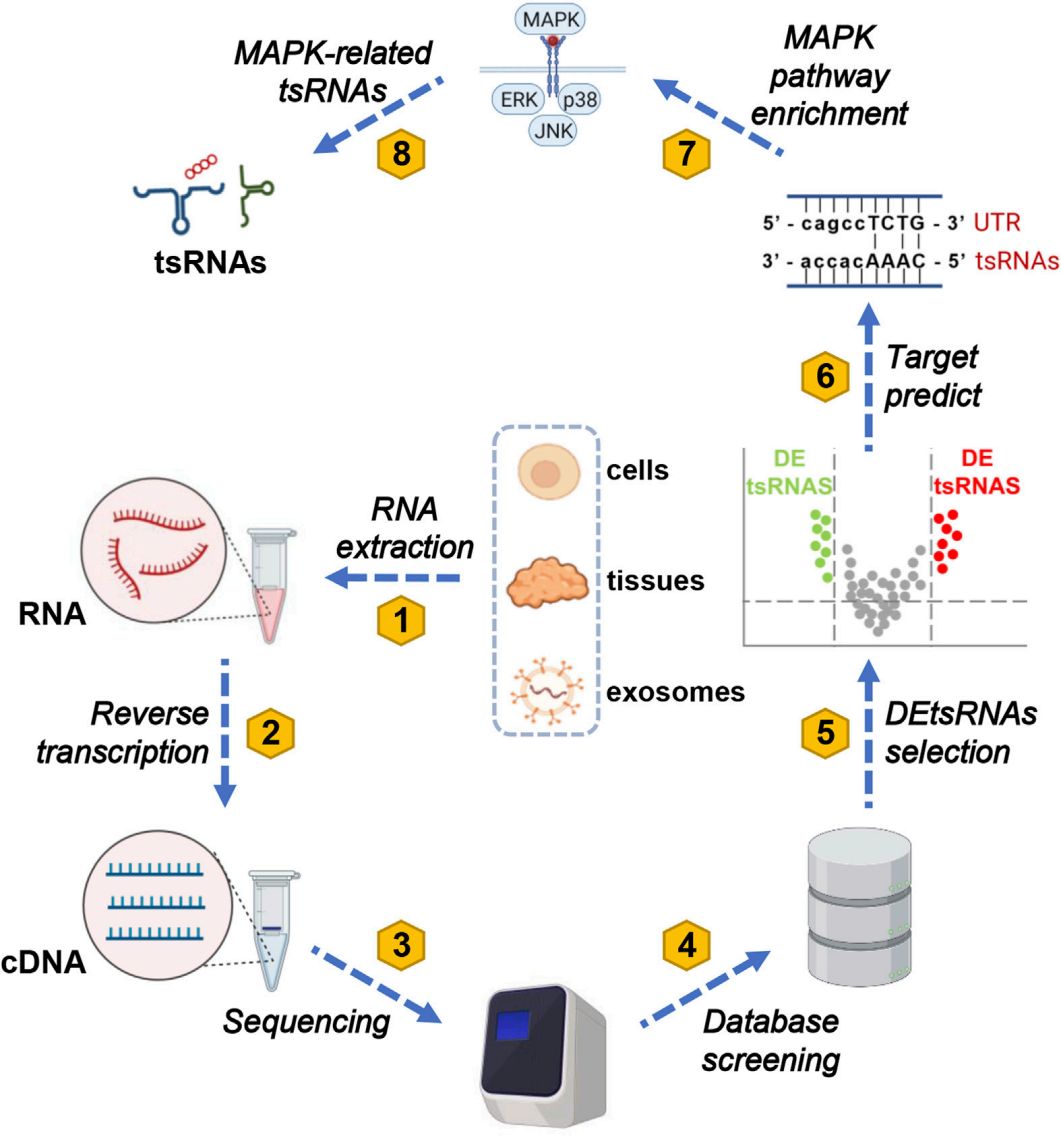
A comprehensive eight-step protocol for MAPK-related tsRNA screening. The screening process for MAPK-related tsRNAs comprises eight distinct steps. Initially, total RNA is extracted from tissues, cells, and exosomes in both diseased and normal groups. Subsequently, this extracted RNA is reverse-transcribed into cDNA. In the third step, the cDNA is submitted to a sequencing platform, where qualified reads are meticulously selected. In the fourth step, these processed reads are meticulously aligned to the genome and cross-referenced with the genomic tRNA database to identify tsRNAs. Moving on to the fifth step, a differential analysis of tsRNAs is conducted to pinpoint differentially expressed tsRNAs (DEtsRNAs). In the sixth step, predictions are made regarding the protein-coding genes (PCGs) downstream of DEtsRNAs, employing tools like TargetScan, RNAhybrid, miRanda, and other relevant websites. In the seventh step, the downstream genes of DEtsRNAs are subjected to KEGG analysis to elucidate the pathways to which they are linked. Finally, this comprehensive approach leads to the identification of MAPK-associated DEtsRNAs within the MAPK signaling pathway. To clarify, in this context, abbreviations such as tsRNAs (tRNA-derived small RNAs), MAPK (mitogen-activated protein kinases), ERK (extracellular-signal-regulated kinase), JNK (c-Jun N-terminal kinase), p38 (38 kDa protein), UTR (untranslated region), tsRNA, and cDNA (complementary DNA) represent key components of the methodology.

GO and KEGG analysis plays a crucial role by offering essential functions such as functional annotation, pathway enrichment, data visualization, and data comparison and integration in subsequent experimental analyses. These tools aid researchers in interpreting experimental results effectively. They facilitate a deeper exploration of gene or protein functions, regulatory mechanisms, biological processes, and the onset and progression of diseases. Moreover, they contribute significantly to shaping the direction of subsequent experiments (Hashimoto et al., 2006). A total of seven tsRNAs related to the MAPK pathway were identified through data mining. The workflow for analyzing these tsRNAs is presented in Figure 2. Current research typically involves extracting RNA from cells, tissues, and exosomes to create corresponding cDNA for sequencing. This data is then compared with various tRF databases, including the Genomic tRNA Database (http://gtrnadb.ucsc.edu) (Fehlmann et al., 2021), tRFdb (http://genome.bioch.virginia.edu/tRFdb) (Kumar et al., 2015), tRFMINTbase (http://cm.jefferson.edu/MINTbase/) (Pliatsika et al., 2018), and NCBI (https://www.ncbi.nlm.nih.gov/) (Kim et al., 2023). Additionally, three online platforms are utilized for predicting target proteins of tsRNAs: TargetScan (https://www.targetscan.org/) (Agarwal et al., 2015), RNAhybrid (http://bibiserv.techfak.unibielefeld.de/rnahybrid/) (Kruger and Rehmsmeier, 2006), and miRanda (http://www.microrna.org/microrna/home.do) (Betel et al., 2008). In most studies, the prediction results from RNAhybrid and miRanda are intersected to obtain potential target proteins for the screened tsRNAs (Yu et al., 2019). A subset of studies instead perform the intersection of prediction results from miRanda and TargetScan (Fang et al., 2021). These predicted target proteins for tsRNAs are then enriched in the MAPK signaling pathway using KEGG analysis (Kanehisa et al., 2021), allowing the identification of MAPK-related tsRNAs.

**FIGURE 3:**
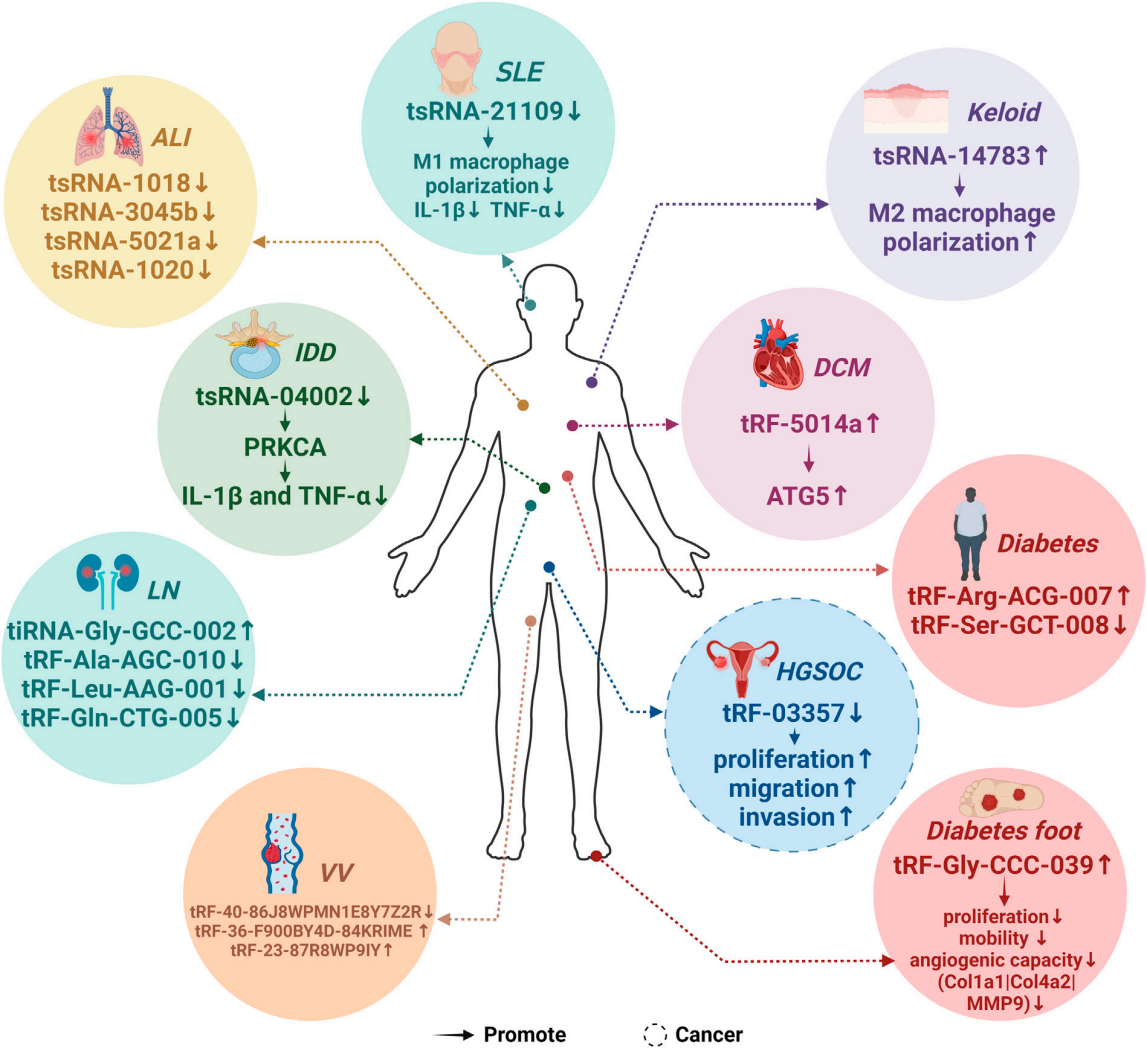
Expression patterns of MAPK-related tsRNAs in diseases. 19 predicted MAPK-related tsRNAs exhibited abnormal expression patterns across 10 distinct diseases. Specifically, the expression of tRF-03357 was notably downregulated in high-grade serous ovarian cancer (HGSOC), whereas tRF-Gly-CCC-039 showed an upregulation in diabetic foot conditions. In diabetes, there is an upregulation in the expression of tRF-Arg-ACG-007, while a downregulation is observed in the expression of tRF-Ser-GCT-008. In varicose veins (VV), both tRF-36-F900BY4D84KRIME and tRF-23-87R8WP9IY exhibited elevated expression levels, while tRF-40-86J8WPMN1E8Y7Z2R demonstrated a significant downregulation. Furthermore, in the context of diabetic cardiomyopathy (DCM), the expression of tRF-5014a was markedly upregulated. Lastly, tsRNA-14783 displayed an upregulation in Keloid cases. The expression of tsRNA-04002 is downregulated in Intervertebral Disc Degeneration (IDD). Similarly, the expression of tsRNA-21109 was downregulated in Systemic Lupus Erythematosus (SLE). Conversely, the expression of tiRNA-Gly-GCC-002 was upregulated in Lupus Nephritis (LN), while the expression of tRF-Ala-AGC-010, tRF-Gln-CTG-005, and tRF-Leu-AAG-001 was downregulated in the same condition. Additionally, in Acute Lung Injury (ALI), the expression of tsRNA-1018, tsRNA-3045b, tsRNA-5021a, and tsRNA-1020 was downregulated.

Figure 3 depicts aberrant tsRNA expression patterns across ten different diseases, Figure 3 illustrates aberrant tsRNA expression patterns across ten distinct diseases, encompassing one cancer and nine non-cancerous conditions. These tsRNAs encompass a range of variants, including tRF-03357 in HGSOC, tRF-36-F900BY4D84KRIME, tRF-23-87R8WP9IY, and tRF-40-86J8WPMN1E8Y7Z2R in VV, tRF-Gly-CCC-039 in diabetic foot, tRF-Arg-ACG-007 and tRF-Ser-GCT-008 in diabetes, tRF-5014a in DCM, tsRNA-14783 in Keloid, tsRNA-04002 in IDD, tsRNA-21109 in SLE, tiRNA-Gly-GCC-002, tRF-Ala-AGC-010, tRF-Gln-CTG-005, and tRF-Leu-AAG-001 in LN, as well as tsRNA-1018, tsRNA-3045b, tsRNA-5021a, and tsRNA-1020 in ALI (Yu et al., 2019; Zhang et al., 2019; Zhao Y. et al., 2022; Wang and Hu, 2022; Zhang et al., 2023)[37373469][37287061][34464838][34923866][34811808][37259935].

Cancer, characterized by malignant tumors, has been the focus of studies revealing the potential of tsRNAs to serve as tumor markers or influence the MAPK pathway, particularly in tumor metastasis (Yuan et al., 2021).

HGSOC, characterized as a highly specific and high-mortality epithelial-mesenchymal transition carcinoma (Xu J. et al., 2022), exhibited elevated tRF-03357 expression in patients when compared to 18 healthy controls (Zhang et al., 2019). KEGG analysis suggested strong associations between tRF-03357 and the MAPK pathway. Experimental results confirmed that reduced tRF-03357 expression hindered HMBOX1 expression, consequently promoting proliferation, migration, and invasion of SK-OV-3 cells (Zhang et al., 2019).

tsRNAs have been identified as influential factors in nine non-cancer diseases through their modulation of the MAPK pathway.

VV denotes vein tortuosity and expansion due to venous factors and blood-related issues (Atkins et al., 2020; Bohler, 2023). Comparison of VV pathological tissues with normal surrounding tissues unveiled upregulation of tRF-36-F900BY4D-84KRIME and tRF-23-87R8WP9IY, alongside downregulation of tRF-40-86J8WPMN1E8Y7Z2R. KEGG analysis tied these findings to the MAPK pathway (Yu et al., 2019).

Diabetes foot, a severe diabetes complication, results from high blood sugar-induced blood vessel and nerve damage in the foot (McDermott et al., 2023). A total of 55 differentially expressed tsRNAs emerged from diabetic foot ulcer tissues, with tRF-Gly-CCC-039 showing high expression and correlation with the MAPK pathway through KEGG analysis. Elevated tRF-Gly-CCC-039 hindered proliferation, motility, angiogenic capacity, and expression of specific genes in human umbilical vein endothelial cells (HUVECs) (Zhang et al., 2023). In diabetes, there is an upregulation in the expression of tRF-Arg-ACG-007, whereas the expression of tRF-Ser-GCT-008 is downregulated. Consequently, these tsRNAs hold promise for identifying potential targets in the diagnosis and treatment of diabetes [37373469].

DCM, a serious diabetes complication causing cardiac issues (Zhao Y. et al., 2022), highlighted four highly expressed tsRNAs (tRF-5014a, tRF-3038b, tRF-3028b/3029b, tRF-5013b) and one downregulated tsRNA (tRF-3009a) in DCM tissues. KEGG analysis revealed a strong link to the MAPK pathway. Notably, tRF-5014a was the most significantly upregulated tsRNA, impacting autophagy and cellular viability (Zhao Y. et al., 2022).

Keloid, an abnormal scar formation (Huang and Ogawa, 2021), showed differential tsRNA expression in M2 macrophages from keloid patients versus adjacent normal tissues. Among them, tsRNA-14783 emerged as significant, possibly influencing keloid formation and development via M2 macrophage polarization (Wang and Hu, 2022).

Intervertebral disk degeneration (IDD) is a degenerative condition underlying various musculoskeletal and spinal disorders. By targeting PRKCA, tsRNA-04002 inhibits the expression of IL-1β and TNF-α and modulates the MAPK signaling pathway, thus suppressing nucleus pulposus cell apoptosis and mitigating IDD [37287061].

Systemic lupus erythematosus (SLE) is a chronic autoimmune disease characterized by M1 macrophage activation. tsRNA-21109 mitigates SLE by inhibiting macrophage M1 polarization, reducing TNF-α and IL-1β expression levels, and modulating the MAPK pathway [34464838]. Lupus nephritis (LN) is a significant end-organ complication of SLE. Upregulated tiRNA-Gly-GCC-002, and downregulated tRF-Ala-AGC-010, tRF-Gln-CTG-005, and tRF-Leu-AAG-001 may contribute to LN pathogenesis by influencing the MAPK pathway’s expression [34923866].

Acute lung injury (ALI) manifests as impaired alveolar function and excessive inflammation. Abnormal downregulation of tsRNA-1018, tsRNA-3045b, tsRNA-5021a, and tsRNA-1020 in ALI patients impacts the MAPK pathway’s expression, thereby ameliorating lung injury, reducing inflammation, and alleviating pulmonary edema [34811808].

## 4 Deciphering MAPK signaling pathways and tsRNAs in cellular processes and tumorigenesis

**FIGURE 4:**
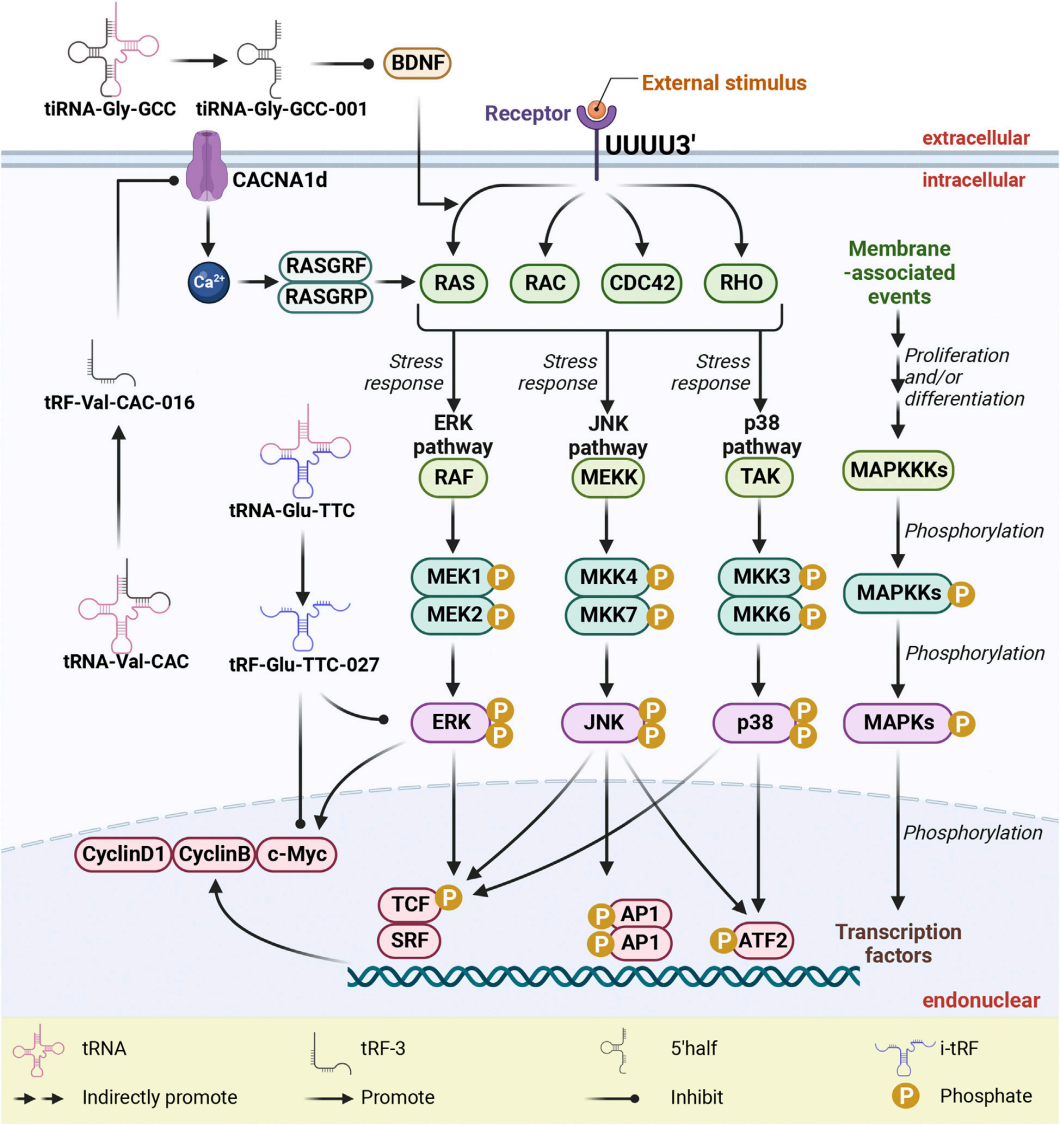
tsRNAs as inhibitors of MAPK pathway activation via targeting MAPK-related proteins. tsRNAs exert their inhibitory influence on the activation of the MAPK pathway through precise targeting of MAPK-related proteins, which notably include BDNF, c-Myc, cyclinB, cyclinD1, ERK, and p38, ultimately leading to a suppression in their expression. To clarify, within this context, abbreviations such as tsRNAs (tRNA-derived small RNAs), tRFs (tRNA-derived fragments), MAPK (mitogen-activated protein kinases), ERK (extracellular-signal-regulated kinase), JNK (c-Jun N-terminal kinase), and p38 (38 kDa protein) are integral to understanding the molecular mechanisms at play.

The MAPK signaling pathway plays a pivotal role in governing fundamental cellular processes, including cell proliferation, differentiation, apoptosis, angiogenesis, and tumor metastasis (Che et al., 2019; Cui et al., 2019; Guo et al., 2020; Yuan et al., 2021; Zhao et al., 2021; Zhao L. et al., 2022). As shown in Figure 4, the regulatory impact of this pathway is substantiated by the involvement of specific RNA fragments, namely, tRF-Glu-TTC-027, tRF-Val-CAC-016, and tiRNA-Gly-GCC-001, which intersect with distinct facets of MAPK activity. In the context of GC cell lines HGC-27 and NCI-N87, tRF-Glu-TTC-027 orchestrates the downregulation of p-p38 and c-Myc, while also targeting p-ERK and p-p38 (Xu et al., 2021). Similarly, tRF-Val-CAC-016 emerges as a modulator by effectively targeting CyclinD1, CyclinB, and c-Myc within GC cell lines NCI-N87 and HGC-27 (Xu W. et al., 2022). Meanwhile, tiRNA-Gly-GCC-001 contributes to the regulatory framework by targeting BDNF (Qin et al., 2019). tRF-Glu-TTC-027 acts as a MAPK inhibitor to inhibit the expression of signaling pathways, thereby inhibiting cell migration and invasion (Xu et al., 2021). tRF-Val-CAC-016 combined with CACNA1d 3 'UTR inhibited the expression of CACNA1d. The decrease of CACNA1d leads to the inhibition of MAPK pathway, resulting in decreased expression of CyclinD1, CyclinB, and c-Myc (Xu W. et al., 2022).

Central to the panoply of MAPK signaling cascades, the RAS/RAF/MEK/ERK pathway assumes paramount significance in the survival and development of tumor cells (Guo et al., 2020). Concomitantly, the RAC/MEKK/MKK/JNK pathway governs cellular stress responses, inflammatory cytokines, and cellular outcomes prompted by environmental stressors [32046099]. Moreover, the RAC or CDC42/TAK/MEK/p38 pathway is closely entwined with cell cycle regulation and apoptosis (Garcia-Hernandez et al., 2021). In the intricate landscape of signal transmission, G-proteins, encompassing RAS, RAC, and CDC42, stand pivotal in signal reception and propagation (Nobes and Hall, 1995; Garcia-Hernandez et al., 2021). Conversely, the MAPKKKs, represented by RAF, MEKK, and TAK, as well as the MAPKKs—MEK and MKK, function as phosphokinases, instigating the downstream phosphorylation cascade (Guo et al., 2020; Garcia-Hernandez et al., 2021). Eliciting a chain reaction, G-proteins activate MAPKKKs, culminating in the phosphorylation of MAPKKs and, ultimately, the activation of MAPKs, ushering their phosphorylation-driven participation in signaling pathways that impinge upon downstream nuclear factors (Garcia-Hernandez et al., 2021). In the realm of GC, the engagement of tRF-Glu-TTC-027 and tRF-Val-CAC-016 is predominantly oriented towards the ERK and p38 pathways, whereas in SCI, tiRNA-Gly-GCC-001 predominantly impacts the ERK pathway.

Among the constituents of the p38 MAPK family—p38α (MAPK14), p38β (MAPK11), p38γ (SAPK3, ERK6, or MAPK12), and p38δ (SAPK4 or MAPK13)—p38 manifests as a seminal entity associated with cell stress and apoptosis (Cuadrado and Nebreda, 2010). Significantly, tRF-Glu-TTC-027 and tRF-Val-CAC-016 exert a potent inhibitory influence on the MAPK signaling pathway by targeting p38, impeding its phosphorylation and thereby incapacitating downstream factors such as p53, Bax, and c-Myc, consequently compromising the apoptotic potential of GC cells (Xu et al., 2021; Xu W. et al., 2022).

Operating as a downstream player within the MAPK signaling cascade, c-Myc emerges as a pivotal transcriptional regulator, intricately steering cell growth, proliferation, and apoptosis (Shi et al., 1993; Fornari et al., 1996; Trudel et al., 1997; Pathria et al., 2021). In a concerted effort, tRF-Glu-TTC-027 and tRF-Val-CAC-016 mediate the inhibition of c-Myc expression, underscoring their role in impeding the orchestration of cellular growth, proliferation, and apoptosis (Xu et al., 2021; Xu W. et al., 2022).

The crux of the MAPK landscape revolves around ERK1/2, a phosphokinase vital to signal transduction that translates extracellular cues into intracellular responses, promoting cell growth while eliciting anti-apoptotic effects (Guo et al., 2020). Herein, tRF-Glu-TTC-027 emerges as a key regulatory element, targeting ERK and thereby suppressing its phosphorylation, effectively stymying the ERK/MAPK signaling axis and obstructing the signal propagation to downstream targets, most notably c-Myc, consequently abrogating the anti-apoptotic impact (Xu et al., 2021).

CACNA1d is a calcium voltage-gated channel subunit located on the cell membrane, which can activate guannylic acid exchange factors such as RasGRF and RasGRPs by increasing intracellular calcium ion concentration, and participate in the activation of Ras. Further activation of MAPK signaling pathway (Fernandez-Medarde and Santos, 2011; Stone, 2011; Xu W. et al., 2022; Ortner, 2023). tRF-Val-CAC-016 combined with CACNA1d 3 'UTR inhibited the expression of CACNA1d. The decrease of CACNA1d leads to the inhibition of MAPK pathway, resulting in the decreased expression of CyclinD1, CyclinB, and c-Myc (Xu W. et al., 2022). Concomitantly, the intricate orchestration of cell cycle progression pivots upon CyclinD and CyclinB, critical factors shaping this regulatory journey (Liang et al., 2019). Evidently, tRF-Val-CAC-016 assumes a pivotal role by selectively targeting CyclinD1 and CyclinB, thereby affecting cytokine transcription, perturbing cell cycle integrity, and inhibiting the genesis of tumors as well as tumor invasiveness (Xu W. et al., 2022).

In parallel, BNDF, a neurotrophic factor, exerts its influence through a sequence of events, involving the exchange of GDP for GTP on Ras protein, culminating in Ras activation. Activated Ras in turn interacts with downstream Raf kinases, subjecting them to phosphorylation by other kinases. This catalytic chain reaction consequently activates MEK 1/2 kinase, propelling the cascade that culminates in the phosphorylation of ERK 1/2 kinase. This activated ERK 1/2 kinase translocates to the nucleus, instigating the phosphorylation of an array of transcription factors, thereby orchestrating gene expression pivotal to cell cycle, proliferation, differentiation, and plasticity (Liu et al., 2021). Pertinently, tiRNA-Gly-GCC-001 intervenes in this intricate interplay by suppressing the expression of the MAPK signaling pathway through BDNF inhibition (Qin et al., 2019).

## 5 Exploring tsRNAs as biomarkers and therapeutic targets in cancer: insights from expression and regulation

The expression of transfer RNA-derived small RNAs (tsRNAs) has emerged as a promising candidate for both potential biomarkers and novel therapeutic targets within the context of cancer. This potential is underscored by compelling findings detailed in Table 1. Notably, aberrantly expressed tsRNAs such as tRF-Glu-TTC-027, tRF-Val-CAC-016, and tRF-24-V29K9UV3IU have been identified in gastric cancer (GC). In a cohort of 33 GC patients, stratified into a high expression group (n = 12) and a low expression group (n = 21) based on tRF-Glu-TTC-027 levels, a comparative analysis revealed that the latter exhibited larger tumor sizes and histological features indicative of poor differentiation. Similarly, using the expression of tRF-Val-CAC-016 as a criterion, 40 patients were categorized into high (n = 15) and low (n = 25) expression groups. Intriguingly, the low expression group of tRF-Val-CAC-016 displayed larger tumor sizes and poor-differentiated histology (Xu et al., 2021; Xu W. et al., 2022).

Furthermore, an array of tsRNAs has been validated as regulators of protein-coding genes (PCGs), potentially influencing the MAPK pathway. This dynamic interplay is highlighted by downregulated tRF-Glu-TTC-027 and tRF-Val-CAC-016 in GC, along with upregulated tRF-03357 in high-grade serous ovarian carcinoma (HGSOC) and tRF-5014a in dilated cardiomyopathy (DCM), as expounded in Table 2. To illustrate, tRF-Glu-TTC-027 binds to the 3 'UTR of TGFB2 and ELK4 (Xu et al., 2021), while tRF-Val-CAC-016 modulates CACNA1d (Xu W. et al., 2022). Similarly, tRF-03357 targets HMBOX1 (Zhang et al., 2019), and tRF-5014a interacts with ATG5 (Zhao Y. et al., 2022). Given the established link between aberrant MAPK signaling and drug resistance across diverse cancers, a potential therapeutic avenue emerges: targeted interventions centered around the downstream PCGs of the tsRNAs/MAPK axis could potentially mitigate the emergence of drug resistance. Encouragingly, Table 2 underscores this potential by revealing several existing drugs, targeting the PCGs downstream of tRFs, which are available in the market. These encompass compounds such as Nimodipine, Ethanol, Felodipine, Nifedipine, and Dronedarone, targeting the downstream effects of CACNA1d, as well as Copper, Chondroitin sulfate, and Esketamine, targeting BDNF downstream of tiRNA-Gly-GCC-001 (Hartung et al., 2022). Notably, further exploration is warranted to elucidate the intricate interactions between the tsRNAs/MAPK axis and these pharmacological agents, as highlighted by this endeavor into the CADDIE database. Dexmedetomidine (DEX) has been found to modulate the expression of the MAPK pathway by targeting specific transfer RNA-derived small RNAs (tsRNAs) such as tsRNA-1018, tsRNA-3045b, tsRNA-5021a, and tsRNA-1020, which are observed to be aberrantly downregulated in patients with Acute Lung Injury (ALI). This mechanism contributes to the improvement of lung injury by mitigating inflammation and reducing pulmonary edema in ALI patients [34811808]. Leveraging DEX’s targeting of tsRNAs to modulate MAPK pathway expression presents a promising avenue for therapeutic intervention [34811808].

**TABLE 2:** The binding sites of tsRNAs and MAPK-related PCGs and the related drugs of MAPK-related PCGs.

tsRNAs	Diseases	Downstream PCGs	Binding site between tRFs and target PCGs		Related drugs (target PCGs)	Ref.
			tsRNAs (3′-5′)	PCGs (5′-3′)		
tRF-Glu-TTC-027	Downregulated in GC	TGFB2	uuUCUUUCCAGGUCAGu	aaAGAAACTTTCAGTCa	—	Xu et al. (2021)
		ELK4	uuucuuucCAGGUCAGu	gaaattcaGTCCAGTCa	—	
tRF-Val-CAC-016	Downregulated in GC	CACNA1d	uuugcCCUUGGUGAa	agcatGAAACCACTt	Nimodipine| Ethanol| Felodipine| Nifedipine| Dronedarone	Xu et al. (2022a)
tRF-03357	Upregulated in HGSOC	HMBOX1	—	—	—	Zhang et al. (2019)
tiRNA-Gly-GCC-001	Upregulated in SCI	BDNF	GACUUGGUGGGUACG	GCG​TGT​GTG​ACA​GTA​TTA​GCG​AG	Copper| Chondroitin sulfate| Esketamine	Qin et al. (2019)
tRF-5014a	Upregulated in DCM	ATG5	ggc​GAU​GUG​A-UAC​CUU​Gg	ctc​CT-CGC​TAG​ATT​GGA​ACc	—	Zhao et al. (2022a)

GC, gastric cancer; HGSOC, high-grade serous ovarian cancer; SCI, spinal cord injury; DCM, diabetic cardiomyopathy; tsRNAs, tRNA-derived small RNAs; tRFs, tRNA-derived fragments; PCGs, protein-coding genes; FZD3, frizzled class receptor 3; VANGL1, Van Gogh-like protein 1; BDNF, brain-derived neurotrophic factor.

## 6 Overview and outlook

Presently, research indicates that tsRNAs predominantly exert their influence on the MAPK signaling pathway through mechanisms such as transcriptional regulation, post-transcriptional processing, and interaction with RNA-binding proteins, akin to the RNA silencing effect observed in miRNAs. However, there remain numerous unexplored aspects concerning other mechanisms through which tsRNAs operate within the MAPK pathway, necessitating further investigation. Firstly, with regard to the transcriptional regulatory mechanisms of tsRNAs in the MAPK pathway, existing studies primarily concentrate on the regulation of MAPK pathway-related gene expression by tsRNAs. It is imperative to delve deeper into understanding how tsRNAs interact with transcription factors or other regulatory elements, thereby influencing the activity and signaling cascades of the MAPK pathway. Secondly, concerning the post-transcriptional processing mechanisms of tsRNAs in the MAPK pathway, current research primarily emphasizes the stability and degradation processes of tsRNAs. A more comprehensive understanding of the processing and modification mechanisms of tsRNAs is necessary. Insight into these processes will illuminate how tsRNAs interact with target genes related to the MAPK pathway. Furthermore, while current research focuses on the interaction between tsRNAs and the AGO protein family, it is crucial to gain a deeper understanding of tsRNAs’ interactions with other RNA-binding proteins. Exploring these interactions will shed light on how tsRNAs modulate the activity and functionality of the MAPK pathway. Future research endeavors should incorporate diverse analytical methods and approaches, such as high-throughput sequencing technology, structural biology, and proteomics. Employing these techniques will aid in uncovering additional mechanisms of tsRNAs in the MAPK pathway, providing a more comprehensive understanding of their regulatory roles.

Currently, research into tsRNAs associated with the MAPK signaling pathway is in its infancy, with studies primarily focusing on the ERK and p38 pathways. However, the MAPK pathway constitutes a vast network, encompassing not only ERK but also JNK, p38, and several sub-pathways like NLK, NIK, IKK, and ERK5. These pathways play pivotal roles in various disease processes such as atherosclerosis, endometrial cancer, and ulcerative colitis (UC) (Shen et al., 2019; Paudel et al., 2021; Dieguez-Martinez et al., 2022). In the future, conducting experiments designed to explore these diverse pathways in the context of specific diseases will facilitate a more comprehensive investigation into tsRNAs related to the MAPK signaling pathway. This approach will enable a thorough understanding of the intricate roles played by tsRNAs in the broader spectrum of MAPK-associated diseases.

In our present investigation, two tsRNAs were found to emulate miRNA-like behavior by curtailing protein phosphorylation. Specifically, tRF-Glu-TTC-027 was observed to target p38, Myc, and ERK, while tRF-Val-CAC-016 exerted its influence on CyclinD1, CyclinB, and c-Myc. However, the intricate mechanistic nuances of tRF-24-V29K9UV3IU and tRF-03357 remain enigmatic, warranting in-depth exploration. Subsequent endeavors should leverage techniques such as RNA-FISH and luciferase reporter gene assays to unravel their precise molecular roles within the MAPK pathway. RNA-FISH, or RNA Fluorescence *In Situ* Hybridization, is a sophisticated technique employed to precisely localize and quantify specific RNA molecules within cells or tissues. By utilizing fluorescently labeled probes that hybridize with target tsRNAs, RNA-FISH enables researchers to visualize the spatial distribution of these molecules with high precision under fluorescence microscopy. This capability is instrumental in elucidating the intricate localization patterns and dynamic alterations of tsRNAs during various cellular processes. On the other hand, the Luciferase Reporter Gene Assay stands as a widely utilized method for analyzing gene expression. This technique involves the construction of a luciferase reporter plasmid, where the regulatory elements responsible for the transcription of the gene of interest are cloned upstream or downstream of the luciferase gene. Subsequently, this plasmid is transfected into cells, and upon appropriate stimulation or treatment, the expression of the target gene is assessed by quantifying luciferase activity. Through this approach, researchers can investigate the impact of tsRNAs on specific gene expression profiles, thereby gaining insights into both the magnitude and modality of their regulatory effects. Presently, the number of samples and pathways studied within the tsRNA/MAPK axis remains limited, with a conspicuous absence of research on the JNK pathway. Future investigations should focus on expanding our understanding of tsRNA impacts on specific MAPK pathways, delving into their unique functionalities and mechanisms. RNA-modifying enzymes possess the ability to add or remove specific modifications, such as methylation or adenosine-to-inosine editing. These enzymes can directly influence tsRNA modifications, altering their structure and function (Kuhle et al., 2023). For instance, AGO proteins typically associate with small RNA molecules like miRNA and siRNA. These small RNAs may harbor nuclear localization signals or enter the nucleus through interactions with other proteins. In certain scenarios, tsRNAs bind with AGO, facilitating their entry into the nucleus (Dou et al., 2019). For tsRNAs whose mechanisms of action have been elucidated, further exploration into the impact of RNA modification and RNA-binding proteins on the biological functions of tsRNAs in specific diseases represents a promising research direction.

MAPK-linked tsRNAs have been associated with a spectrum of non-cancer diseases. For instance, high expression of tRF-36-F900BY4D84KRIME and tRF-23-87R8WP9IY, coupled with low expression of tRF-40-86J8WPMN1E8Y7Z2R, have been linked to elevated VV risk (Yu et al., 2019). Notably, elevated levels of tiRNA-Gly-GCC-001, tRF-Gly-GCC-012, tRF-Gly-GCC-013, and tRF-Gly-GCC-016 have been implicated in modulating the MAPK pathway via BDNF targeting in spinal cord injury (Qin et al., 2019). Furthermore, heightened expression of tRF-Gly-CCC-039 is associated with sustained healing in Diabetic foot cases (Zhang et al., 2023), while tRF-5014a exerts negative regulation on autophagy-related protein ATG5 in DCM (Zhao et al., 2022). The multifaceted roles of tsRNA-14783 in Keloid formation, by steering M2 macrophage polarization, are also being unveiled (Wang and Hu, 2022). While investigations on MAPK-associated tsRNAs in non-cancer ailments remain limited, cardiovascular diseases have witnessed substantial scrutiny, offering a promising avenue for future exploration. In specific diseases like colorectal cancer, angiogenin (ANG) acts as a nuclear nuclease, elevating tRNA expression levels and cleaving the tRNA molecule into 5′ and 3′ ends, leading to an increase in tiRNA levels (Tao et al., 2021). It is well-established that tsRNAs operate through various mechanisms at both pre- and post-transcriptional levels. Besides designing experiments to delve into the mechanisms of abnormally expressed tsRNAs in diseases, exploring alterations in the content of these tsRNAs during disease progression represents a crucial avenue for future research. Understanding these changes in tsRNA content can serve as a valuable research direction, potentially unveiling novel biomarkers.

tsRNAs also exert their influence on MAPK-related pathways, exemplified by tRF-Glu-TTC-027’s involvement in the Wnt pathway. Activation of the Wnt/PCP pathway necessitates MAPK-linked Rac1 protein activation, where tRF-Glu-TTC-027’s targeting of TGFβ2 and ELK4 assumes significance, given TGFβ2’s role in the Wnt/β-catenin signaling cascade (Cajanek et al., 2013). Moreover, emerging MAPK-enriched tsRNAs, such as tRF-03357 in HGSOC, tRF-24-V29K9UV3IU in GC, and tDR-012842 in chronic kidney disease (CKD), underscore the intricate web of interactions. tRF-03357 demonstrates a downregulatory effect on the levels of the inflammatory factor homeobox containing 1 (HMBOX1) in ovarian cancer cell lines SK-OV-3. This downregulation consequently facilitates cell proliferation and movement, as evidenced by previous studies [31,37206587]. Additionally, tDR-012842 has been observed to inhibit the differentiation and maturation of CKD podocyte differentiation by downregulating fibroblast growth factor 10 (FGF10) (Shi et al., 2020)., remain subject to ongoing scrutiny. To unravel the precise regulatory roles and sites of these tsRNAs within the MAPK pathway, comprehensive investigations are imperative. To explore the modulation of tsRNA molecules during the activation of the MAPK pathway, employing high-throughput sequencing technology can furnish a detailed intracellular tsRNA profile. Subsequent bioinformatics analyses enable the prediction of potential target genes and elucidation of tsRNA mechanisms of action. To ascertain the precise involvement of tsRNAs in the MAPK pathway, targeted knockdown or overexpression experiments can be devised. Such experiments offer insights into how tsRNAs impact cell signaling and influence critical MAPK pathway components like MAPKKK, MAPK, MEK, and ERK. Concurrently, *in vivo* investigations are indispensable. The construction of animal models facilitates the observation of tsRNA functions and effects under both physiological and pathological contexts. By comparing phenotypic disparities between wild type and models with knocked out or overexpressed tsRNAs, a more accurate understanding of tsRNA regulatory roles within the MAPK pathway emerges. Moreover, translating these discoveries into clinical applications necessitates exploring the feasibility of tsRNAs as potential therapeutic targets and biomarkers. This entails developing interventions targeting specific tsRNAs and evaluating their clinical relevance in disease diagnosis and prognostic assessment. In summary, unraveling the precise regulatory roles and mechanisms of tsRNAs within the MAPK pathway demands interdisciplinary collaboration and the comprehensive utilization of diverse research methodologies. Such endeavors contribute to a deeper comprehension of RNA regulatory networks in cellular signaling and offer novel strategies for disease diagnosis and treatment.

Given tsRNAs’ ability to induce RNA silencing, thereby influencing phosphorylation and protein expression in MAPK pathway-related proteins, it follows that MAPK-associated tsRNAs could potentially shape cancer cell drug resistance. Nevertheless, numerous tsRNAs remain undiscovered and understudied, and the precise mechanisms of action for many tsRNAs have not been thoroughly investigated. Specifically, the mechanisms underlying MAPK-associated tsRNAs, in comparison to Wnt-associated tsRNAs, remain largely unexplored. A comprehensive understanding of the distinct mechanisms employed by MAPK-associated tsRNAs holds immense potential, particularly in the context of anti-cancer resistance marker research. Currently, drugs targeting non-coding RNAs, such as miRNA-targeting drugs, have been successfully developed and applied in various related diseases. Examples include Miravirsen, an anti-hepatitis C virus drug that targets and inhibits miR-122 (Gebert et al., 2014), and MRG-106, a drug targeting B-cell lymphoma that inhibits miR-155 (Seto et al., 2018). Additionally, anti-miRNA drugs like AntagomiRs (Innao et al., 2020) have been utilized to counteract specific miRNA functions, showing promising outcomes in the treatment of diverse diseases, including cancer, cardiovascular disorders, and inflammatory conditions. Given these advancements, there is an urgent need for the development of tsRNA-related drugs. Exploring and harnessing the therapeutic potential of tsRNAs could revolutionize the landscape of disease treatment and pave the way for innovative therapeutic interventions. Future inquiries should scrutinize drug resistance-linked tsRNAs by juxtaposing differentially expressed MAPK-associated tsRNAs in drug-resistant versus non-resistant cancer cell lines. Importantly, constructing drug-resistant cell lines should mirror typical clinical combined chemotherapy regimens. Subsequent experimental investigations, such as transfection studies, could affirm the impact of drug resistance-associated tsRNAs on cancer’s malignant phenotype. In clinical practice, detection of these tsRNA drug resistance markers could serve as invaluable guides for physicians in tailoring effective drug regimens.

## 7 Conclusion

Our study presents a comprehensive overview of the current landscape of regulatory interactions between tsRNAs and the MAPK pathway, shedding light on their significant involvement in cancer development. Concurrently, we compile a comprehensive array of assays employed across numerous studies for the identification of MAPK-associated tsRNAs. Moreover, our investigation illuminates existing limitations within the realm of current research and underscores principal avenues for future exploration in the domain of MAPK-related tsRNAs. In summation, our findings collectively highlight the potential of MAPK-associated tsRNAs as emerging markers for cancer diagnosis, poised to assume a pivotal role in shaping clinical treatment strategies.

## Figures and Tables

**Table:** 

**ALI**	Acute lung injury
**ANG**	angiogenin
**BDNF**	brain-derived neurotrophic factor
**cDNA**	complementary DNA
**CKD**	chronic kidney disease
**CRC**	colorectal cancer
**Cyt c**	cytochrome c
**DCM**	diabetic cardiomyopathy
**DEtsRNA**	differently expressed tsRNA
**eIF**	eukaryotic initiation factor
**ERK**	extracellular-signal-regulated kinase
**FISH**	fluorescent *in situ* hybridization
**FOXK1**	forkhead box K1
**FZD3**	frizzled class receptor 3
**GC**	gastric cancer
**HGSOC**	high-grade serous ovarian cancer
**hnRNP**	heterogeneous nuclear ribonucleoprotein
**HUVECs**	human umbilical vein endothelial cells
**IDD**	Intervertebral disk degeneration
**JNK**	c-Jun N-terminal kinase
**LN**	Lupus nephritis
**MAPK**	mitogen-activated protein kinase
**mRNA**	messenger ribonucleic acid
**p38**	38 kDa protein
**PCGs**	protein-coding genes
**pre-tRNA**	precursor tRNA
**SAPK**	stress-activated protein kinase
**SCI**	spinal cord injury
**SLE**	Systemic lupus erythematosus
**snRNA**	small non-coding RNA
**tRFs**	tRNA-derived fragments
**tiRNA**	tRNA-derived stress-induced RNA
**tsRNAs**	tRNA-derived small RNAs
**UTR**	untranslated region
**VANGL1**	Van Gogh-like protein 1
**VV**	varicose vein
**YBX1**	Y-box binding protein 1
